# A miR-210-3p regulon that controls the Warburg effect by modulating HIF-1α and p53 activity in triple-negative breast cancer

**DOI:** 10.1038/s41419-020-02952-6

**Published:** 2020-09-09

**Authors:** Ye Du, Na Wei, Ruolin Ma, Shuheng Jiang, Dong Song

**Affiliations:** 1 Departments of Breast Surgery, The First Hospital of Jilin University, 130021 Changchun, Jilin P.R. China; 2 State Key Laboratory of Oncogenes and Related Genes, Shanghai Cancer Institute, Ren Ji Hospital, School of Medicine, Shanghai Jiao Tong University, 200240 Shanghai, P.R. China

**Keywords:** Cancer metabolism, Diseases

## Abstract

Reprogrammed energy metabolism, especially the Warburg effect (aerobic glycolysis), is an emerging hallmark of cancer. Different from other breast cancer subtypes, triple-negative breast cancer (TNBC) exhibits high metabolic remodeling, increased aggressiveness and lack of targeted therapies. MicroRNAs (miRNA) are essential to TNBC malignant phenotypes. However, little is known about the contribution of miRNA to aerobic glycolysis in TNBC. Through an integrated analysis and functional verification, we reported that several miRNAs significantly correlates to the Warburg effect in TNBC, including miR-210-3p, miR-105-5p, and miR-767-5p. Ectopic expression of miR-210-3p enhanced glucose uptake, lactate production, extracellular acidification rate, colony formation ability, and reduced serum starvation-induced cell apoptosis. Moreover, GPD1L and CYGB were identified as two functional mediators of miR-210-3p in TNBC. Mechanistically, miR-210-3p targeted GPD1L to maintain HIF-1α stabilization and suppressed p53 activity via CYGB. Ultimately, miR-210-3p facilitated aerobic glycolysis through modulating the downstream glycolytic genes of HIF-1α and p53. Taken together, our results decipher miRNAs that regulate aerobic glycolysis and uncover that miR-210-3p specifically contributes to the Warburg effect in TNBC.

## Introduction

Breast cancer is one of the most common malignancies in women across nations and races. In clinical, the expression status of progesterone receptor, estrogen receptor, and human epidermal growth factor receptor 2 (HER2, overexpression and/or amplification) is of great importance for the management of breast cancer patients. Effective tailored therapies have been developed for breast cancers positive of hormone receptor or HER2 expression^1^. Specifically, triple-negative breast cancer (TNBC) refers to certain breast cancer lack of expression of progesterone receptor, estrogen receptor, and HER2^2^. TNBC is not only resistant to traditional hormone-based therapy and antibody-based therapy, but also associated with higher aggressiveness, frequent distant metastasis, and poor clinical outcome^3^. Therefore, a better understanding of the molecular mechanisms underlying TNBC development and progression is urgently needed.

Reprogrammed energy metabolism is one of hallmarks of cancers^4^. A cancer cell exhibits glycolysis instead of oxidative phosphorylation even in the presence of oxygen, a phenomenon called aerobic glycolysis, also known as Warburg effect. Emerging evidence reveals that glycolysis can be hijacked by cancer cells to promote survival, growth, metastasis, stemness, drug resistance, long-term maintenance, and immune evasion^5–7^. Previously, several studies have well documented the transcriptional regulators of aerobic glycolysis in cancers, such as HIF-1α, c-MYC, p53, FOXK1/2, and SIX1^5,8–12^. However, little is known about the regulatory mechanism of aerobic glycolysis at the post-transcriptional level, such as microRNA (miRNA).

MicroRNAs are small non-coding RNAs of ~22 nucleotides that exhibit significant regulatory roles by post-transcriptionally targeting mRNAs^13^. MicroRNA plays a broad range of oncogenic functions in TNBC including cell proliferation, apoptosis, migration, metastasis, epithelial mesenchymal transition (EMT) process, and angiogenesis^14^. For example, miR551b-3p is aberrantly expressed in TNBC and targeting miR-551b with anti-miR551b-3p reduces tumor growth and metastasis^15^. Additionally, miR-221 transfer from TNBC cells via microvesicles induces EMT and promotes the malignant potential of recipient cells^16^. Recently, several miRNAs have been reported to play a role in regulating the glycolytic phenotypes of breast cancer cells. For instance, microRNA-27b reduces mitochondrial oxidation and promotes extracellular acidification via targeting PDHX, resulting in breast cancer progression^17^; microRNA-155 regulates glucose metabolism through targeting PIK3R1-FOXO3a-cMYC axis in breast cancer^18^; and miR-186-3p/EREG axis orchestrates aerobic glycolysis in ER-positive breast cancer^19^. However, much about miRNA and aerobic glycolysis in TNBC remains to be investigated.

In this study, we comprehensively characterized miRNA related to aerobic glycolysis in TNBC through leveraging large-scale TNBC molecular profiles from The Cancer Genome Atlas (TCGA). Functional verification revealed that miR-210-3p acts as a key regulon that facilitates aerobic glycolysis of TNBC cells via targeting GPD1L and CYGB. This work sheds light on the miRNA-mediated regulatory mechanisms by which TNBC acquires glycolytic propensity to support tumor progression.

## Materials and methods

### Data mining and group information

The RNA-sequencing data of triple-negative breast cancer and corresponding non-tumor tissues were downloaded from The Cancer Genome Atlas (TCGA, https://gdc.cancer.gov/) database. A glycolysis score based on the expression level of glycolytic components (SLC2A1, HK2, GPI, PFKL, ALDOA, PGK1, PGAM1, ENO1, PKM2, and LDHA) was calculated for definition of glycolysis status: high glycolysis (*n* = 57) or low glycolysis (*n* = 56). Differentially expressed miRNA related to glycolysis status was identified by estimating an exact test *P*-value.

### Cell culture

The immortalized human mammary epithelial cell lines MCF10A and human breast cancer cell lines (MCF7, T47D, ZR-75-1, Hs578T, MDA-MB-21, and HCC1937) were all purchased from Cell Resource Center of Shanghai Institutes for Biological Sciences, Chinese Academy of Sciences (Shanghai, China). All cell lines were authenticated and tested for mycoplasma contamination. RPMI-1640 (Hyclone, USA) and Dulbecco’s modified Eagle’s medium (Hyclone, USA) supplemented with 10% fetal bovine serum (Gibco, USA), 100 U/mL penicillin, and 100 μg/mL streptomycin (Invitrogen, USA) were used for cell culture. All cells were cultured in a culture incubator with 5% CO_2_ at 37 °C.

### Cell transfection

The miR-210-3p, miR-105-5p, and miR-767-5p mimics and the negative control duplex (which was non-homologous to all human gene sequences) were used for functional analysis. All the RNA duplexes were synthesized by GenePharma Inc (Shanghai, China). The miRNA Transfection X-treme GENE Reagent (Roche, USA) was used for transient transfection following the manufacturer’s instructions. Overexpression of GPD1L and CYGB was generated by transfection of pcDNA3.1-GPD1L and pcDNA3.1-CYGB plasmids using Lipofectamine 2000 (Invitrogen, USA); empty control plasmids were used to generate control lines.

### Quantitative real-time PCR

Total RNA from breast cancer cells and TNBC samples was extracted by the RNAiso Plus kit (Takara Bio Inc., Japan). Twenty-two TNBC specimens were also obtained from Departments of Breast Surgery, The First Hospital of Jilin University. All the patients were provided with written informed consent before enrollment, and the study was approved by the Research Ethics Committee of The First Hospital of Jilin University. The RNA concentration and quality were determined by spectrophotometry using NanoDrop™ 2000 (Thermo Scientific, USA). For miRNA detection, miRNA was reversely transcribed by the TaqMan microRNA Reverse Transcription kit, and quantitative real-time PCR was performed following the manufacturer’s instructions. U6 RNA was set as an internal control. For mRNA detection, 1 μg of total RNA was reversely transcribed by primeScript RT Master kit (Takara Bio Inc., Japan). Quantitative real-time PCR was performed with SYBR Green using the ViiA7 System (AB Applied Biosystems, USA). The primers used in this study were all available at PrimerBank (https://pga.mgh.harvard.edu/primerbank/).

### Western blotting analysis

For protein preparation, cells were collected with lysis buffer containing 0.1% Triton X-100, 20 mM Tris-Cl, 125 mM NaCl, 0.5 mM EDTA, 1 mM dithiothreitol (DTT), and protease inhibitor cocktail on ice for 15 min. Protein concentration was determined using Pierce BCA Protein assay kit (Thermo Fisher Scientific, USA). Protein samples were separated using 6–10% sodium dodecyl sulfate-polyacrylamide gels and transferred onto polyvinylidene difluoride (PVDF) membranes (Millipore, USA). After blocking with 5% non-fat milk, the membranes were incubated overnight at 4 °C with primary antibodies against GPD1L (17263-1-AP, ProteinTech), CYGB (ab52662, Abcam), FGFRL1 (ab95940, Abcam), HIF-1α (#36169, Cell Signaling Technology), Hydroxy-HIF-1α (Pro564) (#3434, Cell Signaling Technology), and p53 (#2527, Cell Signaling Technology). Anti-β-actin antibody (ab8227, Abcam) was used as an internal control. The next day, the membranes were probed with a horseradish peroxidase (HRP)-conjugated secondary antibody for 1 h at room temperature. Finally, blots were developed using the ECL kit (Millipore, USA).

### Glucose and lactate assay

Glucose uptake and lactate production were used to determine cellular glycolytic activity as reported previously^20^. In brief, 1 × 10^6^ MDA-MB-231 and Hs578T cells were seeded in 60-mm plates and cultured with FBS-free medium. Twenty-four hours later, culture medium was collected and subjected for glucose and lactate analysis using a glucose assay kit (Sigma-Aldrich, Shanghai, China) and a Lactate Assay Kit (BioVision, USA) according to the manufacturer’s instruction. The glucose and lactate levels were normalized to total cell protein.

### Seahorse analysis

Extracellular acidification rate (ECAR) was detected using a Seahorse XF96 analyzer (Seahorse Biosciences, USA). In brief, MDA-MB-231 and Hs578T cells at a density of 20,000 cells per well were seeded in a 96-well cell culture XF96 microplate (Seahorse Biosciences, USA). Before experiments, MDA-MB-231 and Hs578T cell culture medium was replaced and cells were then incubated with assay medium for 1 h at 37 °C in a CO_2_-free incubator. ECAR was detected using sequential injection of 10 mM glucose, 1 mM oligomycin (Sigma-Aldrich) and 50 mM 2-deoxyglucose (2-DG, Sigma-Aldrich). All measurements were recorded at set time intervals (7 min). ECAR after oligomycin treatment indicates glycolytic capacity.

### Luciferase reporter assay

The binding of miR-210-3p to GPD1L or CYGB mRNA in MDA-MB-231 and Hs578T cells was verified using a luciferase reporter assay. The putative miR-210-3p complementary site in the 3′-UTR of GPD1L or CYGB mRNA or its mutant sequence was cloned into the p-MIR-reporter vector (Ambion, USA). For luciferase reporter assay, MDA-MB-231 and Hs578T cells were seeded in 96-well plates and then co-transfected with p-MIR-reporter vectors with miR-210-3p mimics or control mimics using Lipofectamine 2000 (Invitrogen, USA). After transfection for 48 h, the relative luciferase activity was detected using a dual luciferase reporter assay system (Promega, USA).

### Colony formation assay

MDA-MB-231 and Hs578T cells were seeded in six-well plates at 1000 cells per well. Cells were allowed to culture for 10–14 days, then fixed with 4% paraformaldehyde and stained with 0.5% crystal violet staining. Visible colonies were counted.

### CCK-8 assay and cell apoptosis assay

For CCK-8 experiment, MDA-MB-231 and Hs578T cells were seeded in 96-well plates at 3000 cells per well. At indicated time points, cell viability was measured by CCK-8 assay according to the manufacturer’s instructions (Dojindo, Japan). For cell apoptosis, MDA-MB-231 and Hs578T cells were seeded in 96-well plates at 5,000 cells per well. After serum starvation for 48 h, cell apoptosis was detected by Apo-ONE Caspase-3/7 assay (Promega, G7790) according to the manufacturer’s instructions.

### Animal experiment

Balb/c nude mice (male, 6 weeks) were manipulated and housed according to the criteria outlined in the “Guide for the Care and Use of Laboratory Animals” prepared by the National Academy of Sciences. Mice were kept on a 12-hour day/night cycle with free access to food and water. Mice were divided into two groups at random. The investigator was blinded to the group allocation during the experiment. For generation of subcutaneous xenograft model, 2 × 10^6^ control or miR-210-3p-overexpressing MDA-MB-231 cells were suspended in PBS and injected subcutaneously in the lower back. Three weeks later, mice were sacrificed and tumor weight was calculated. This study was approved by the Research Ethics Committee of The First Hospital of Jilin University.

### HRE luciferase reporter assay

At 24 h before miR-210-3p mimics or control mimics transfection, pcDNA3.1-vector and GPD1L-overexpressing MDA-MB-231 and Hs578T cells were transfected with plasmids encoding HRE-firefly luciferase. After transfection for 48 h, luciferase activities were evaluated using a dual luciferase reporter assay system (Promega, USA). The HRE activity was shown as firefly luciferase counts relative to Renilla luciferase counts.

### Statistical analysis

Data were presented as the means ± SD. The statistical analysis was performed with GraphPad Prism 5 (GraphPad Software, San Diego, CA, USA). The two-sided Student’s t test or one-way ANOVA followed by Student-Newman-Keuls (SNK) test was used to compare data between groups. For all tests, a p-value of less than 0.05 was considered statistically significant.

## Results

### Integrated analysis of key miRNAs involved in the Warburg metabolism of TNBC

**Fig. 1:**
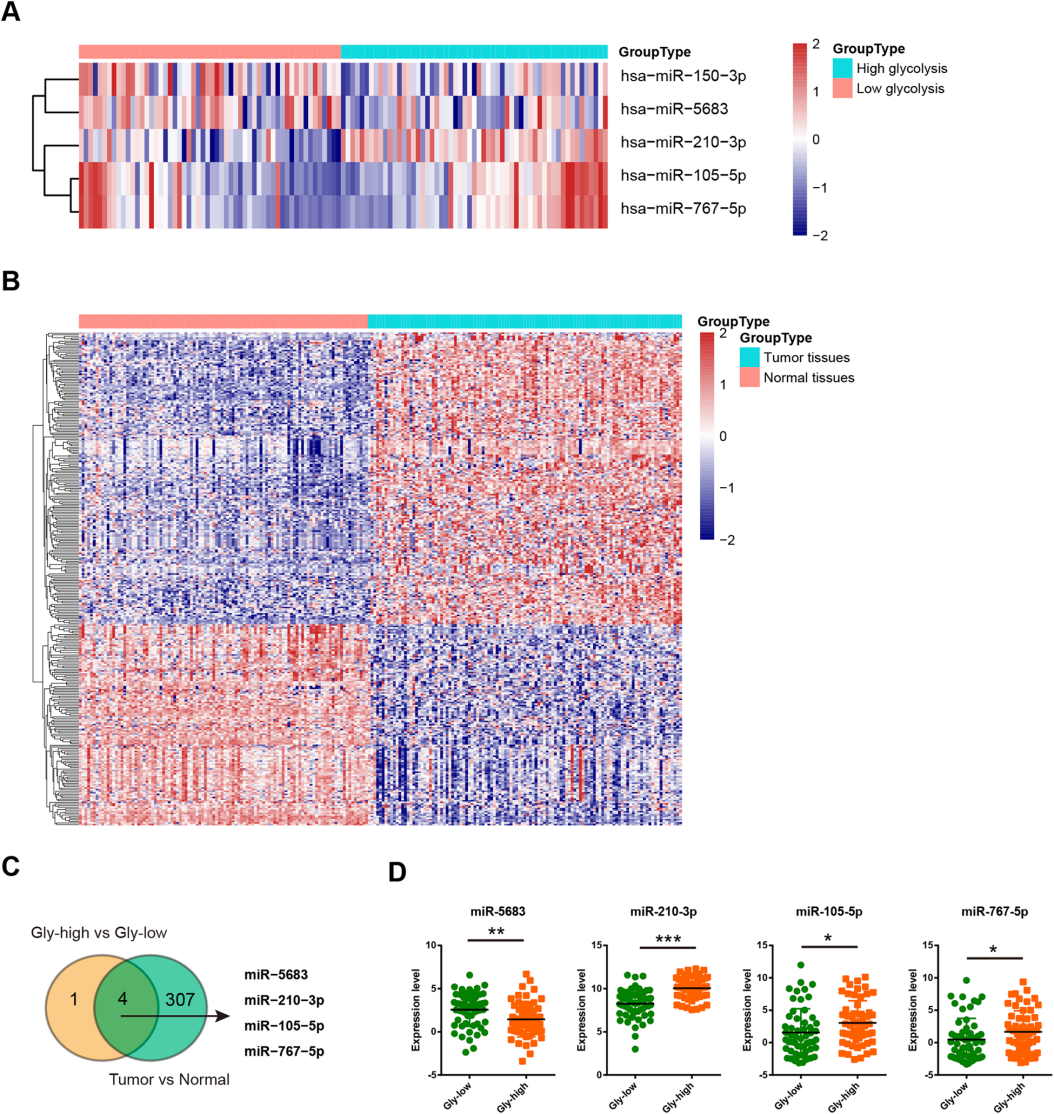
Integrated analysis of key miRNAs involved in the Warburg metabolism of TNBC. **a** Heatmap of 5 miRNAs related to TNBC glycolysis. **b** Heatmap of differentially expressed miRNAs in TNBC tumor tissues compared with matched normal tissues. **c** Venn diagram showed that four glycolysis-related miRNAs were upregulated or downregulated in TNBC tissues. **d** Expression level of miR-5683, miR-210-3p, miR-105-5p, and miR-767-5p in high glycolysis group and low glycolysis group. *p < 0.05; **p < 0.01; ***p < 0.001.

To identify which miRNAs contribute to the glycolytic phenotype in TNBC, data from triple-negative breast invasive carcinoma patient samples were acquired from The Cancer Genome Atlas (TCGA) and stratified into high glycolysis group (*n* = 57) or low glycolysis group (*n* = 56) based on a glycolysis score generated from expression of 10 known glycolytic components (Supplementary Table 1). By comparing the differentially expressed miRNA, five miRNAs (miR-150-3p, miR-5683, miR-210-3p, miR-105-5p, and miR-767-5p) had at least two-fold change in the high glycolysis subtype compared with the low glycolysis subtype (Fig. 1a). We also identified that 311 miRNAs were differentially expressed in TNBC tissues compared with their normal counterparts (Fig. 1b). Of note, four glycolysis-related miRNAs were dysregulated (Fig. 1c). Specifically, miR-5683 expression was downregulated, while miR-210-3p, miR-105-5p, and miR-767-5p were upregulated in TNBC tissues (Fig. 1d).

### miR-210-3p, miR-105-5p, and miR-767-5p promote aerobic glycolysis in TNBC cells

**Fig. 2:**
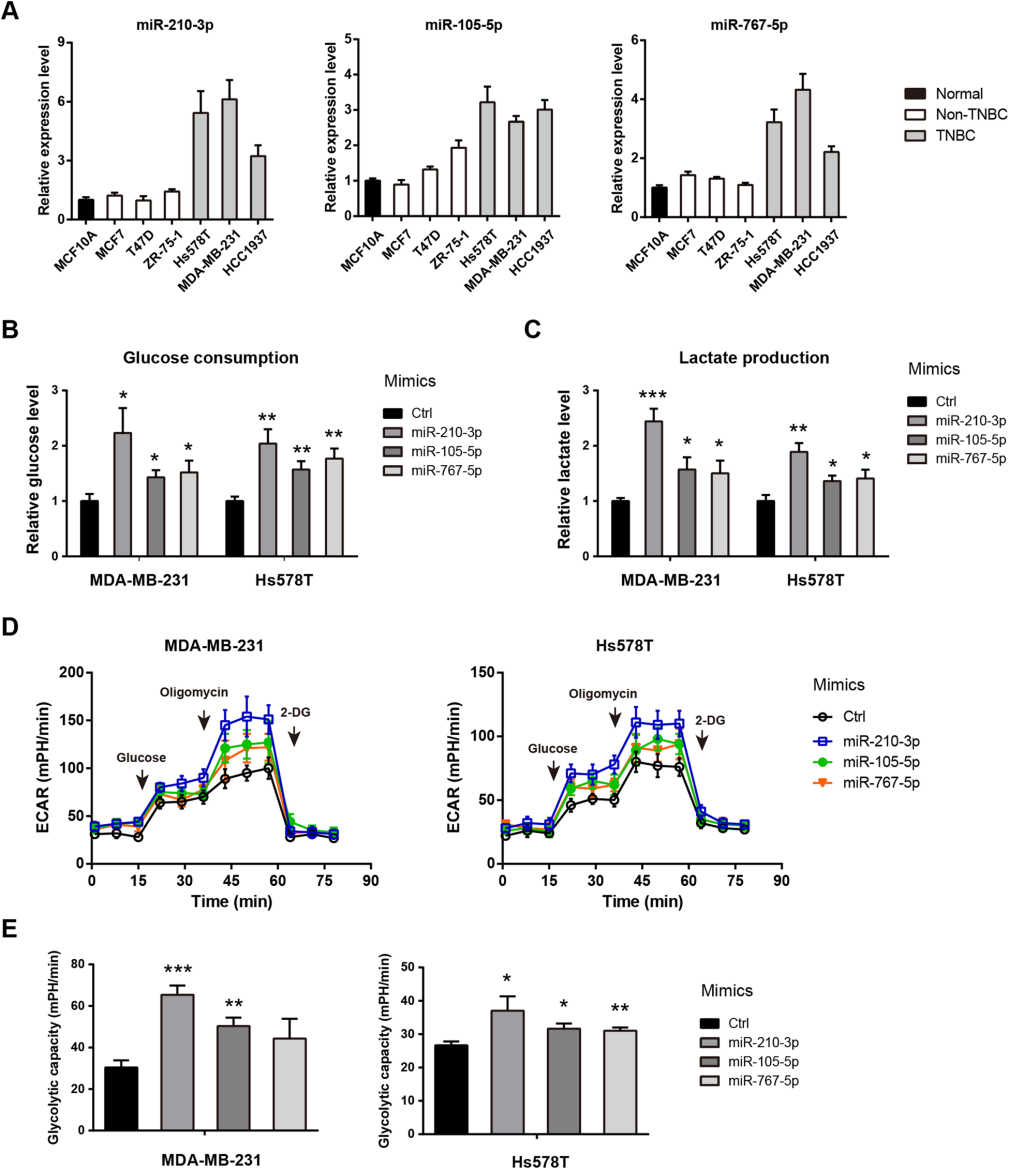
miR-210-3p, miR-105-5p, and miR-767-5p promote aerobic glycolysis in TNBC cells. **a** Real-time qPCR analysis of miR-210-3p, miR-105-5p, and miR-767-5p expression in breast cancer cells. **b**–**e** Quantification of glucose consumption (**b**), lactate production (**c**), extracellular acidification rate (**d**), and glycolytic capacity (**e**) in MDA-MB-231 and Hs578T cells after transfection of miR-210-3p, miR-105-5p, miR-767-5p mimics and control mimics. *p < 0.05; **p < 0.01; ***p < 0.001.

Next, we determine whether the highly expressed miRNAs (miR-210-3p, miR-105-5p, and miR-767-5p) are implicated in the glycolytic metabolism in TNBC. To achieve this, we first evaluated their expression in breast cancer cell lines. As showed in Fig. 2a, miR-210-3p, miR-105-5p, and miR-767-5p were highly expression in TNBC cells (Hs578T, MDA-MB-231, and HCC1937) compared to non-TNBC cells (MCF7, T47D, and ZR-75-1) and the immortalized breast epithelial cell MCF-10A. Then, MDA-MB-231 and Hs578T cell lines were selected for cellular function verification by transfection of miR-210-3p, miR-105-5p, miR-767-5p mimics, and control mimics. Cellular glycolytic activity was determined by glucose uptake and lactate production. As a result, miR-210-3p, miR-105-5p, and miR-767-5p significantly and differentially increased glucose uptake (Fig. 2b) and lactate production (Fig. 2c) in MDA-MB-231 and Hs578T cells. To further confirm this observation, a Seahorse XF96 analyzer was used. Consistently, the extracellular acidification rate (Fig. 2d) and glycolytic capacity (Fig. 2e) was upregulated by miR-210-3p, miR-105-5p, and miR-767-5p in MDA-MB-231 and Hs578T cells. Notably, miR-210-3p had a maximum stimulatory effect in promoting TNBC aerobic glycolysis. Therefore, miR-210-3p was selected for further molecular mechanism study.

### miR-210-3p directly targets GPD1L and CYGB in TNBC

**Fig. 3:**
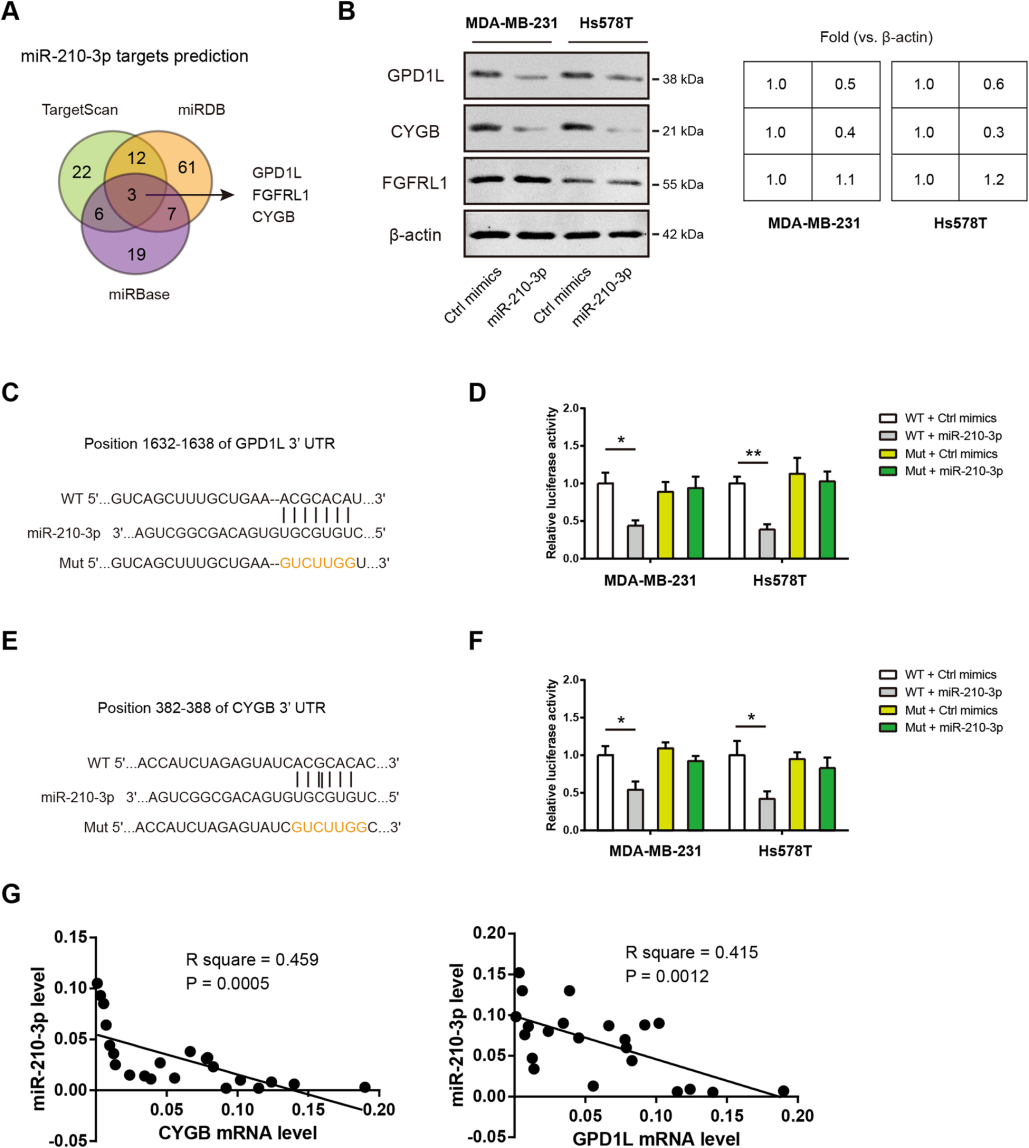
miR-210-3p directly targets GPD1L and CYGB in TNBC. **a** Predictive target genes of miR-210-3p from TargetScan, miRDB, and miRBase. **b** Western blotting analysis of GPD1L, CYGB and FGFRL1 protein expression in MDA-MB-231 and Hs578T cells after transfection of miR-210-3p; β-actin was loaded as an internal control. **c** Predicted miR-210-3p target sequences in 3′-UTR of GPD1L; a mutant 3’UTR with substitutions in the complementary site for the seed region of miR-210-3p was used as a control. **d** Luciferase reporter activity in MDA-MB-231 and Hs578T cells co-transfection of the luciferase reporter vector (GPD1L-WT or GPD1L-Mut) and miR-210-3p mimics; luciferase activity was measured 48 h after transfection. **e** Predicted miR-210-3p target sequences in 3′-UTR of CYGB; a mutant 3′-UTR with substitutions in the complementary site for the seed region of miR-210-3p was used as a control. **f** Luciferase reporter activity in MDA-MB-231 and Hs578T cells co-transfection of the luciferase reporter vector (CYGB-WT or CYGB-Mut) and miR-210-3p mimics; luciferase activity was measured 48 h after transfection. **g** Correlation analysis between miR-210-3p and CYGB/GPD1L expression in 22 TNBC samples. *p < 0.05; **p < 0.01.

To uncover the molecular targets of miR-210-3p in TNBC, we used the publicly available algorithms TargetScan, miRDB, and miRBase to predict the potential targets. The result showed that GPD1L, FGFR1, and CYGB, may be potential targets of miR-210-3p (Fig. 3a). Western blotting analysis showed that miR-210-3p can reduce the protein level of GPD1L and CYGB but not FGFRL1 in MDA-MB-231 and Hs578T cells, indicating that GPD1L and CYGB may act as downstream targets of miR-210-3p (Fig. 3b). To test this possibility, we constructed a p-MIR-reporter containing the complementary seed sequence of miR-210-3p in the 3’-UTR region of GPD1L or CYGB mRNA and a control reporter containing a mutated sequence of the same fragment (Fig. 3c, e). Then, luciferase reporter assay revealed that miR-210-3p repressed the reporter activity driven by the 3’-UTRs of GPD1L or CYGB in MDA-MB-231 and Hs578T cells; in contrast, luciferase activities were unaffected in the mutant form (Fig. 3d, f). In clinical TNBC samples, a significant inverse correlation was revealed between miR-210-3p and these two molecular targets (Fig. 3f). Collectively, these findings suggest that GPD1L and CYGB are direct targets of miR-210-3p in TNBC.

### Ectopic expression of GPD1L or CYGB suppresses aerobic glycolysis in TNBC cells

**Fig. 4:**
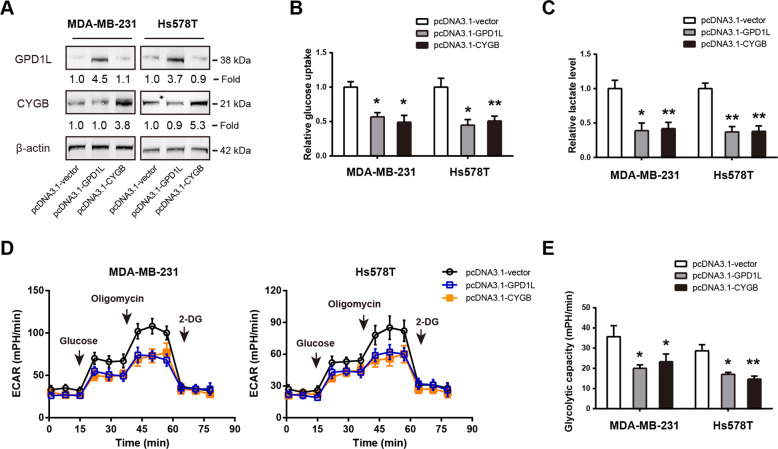
Ectopic expression of GPD1L or CYGB suppresses aerobic glycolysis in TNBC cells. **a** Western blotting analysis of the overexpression efficiency of GPD1L or CYGB in MDA-MB-231 and Hs578T cells. **b**–**e** Quantification of glucose consumption (**b**), lactate production (**c**), extracellular acidification rate (**d**), and glycolytic capacity (**e**) in MDA-MB-231 and Hs578T cells after ectopic expression of GPD1L or CYGB. *p < 0.05; **p < 0.01.

To investigate whether the targets of miR-210-3p, GPD1L and CYGB, play a role in the glycolytic phenotype of TNBC cells, we performed gain-of-function experiments. The overexpression efficiency of GPD1L and CYGB in MDA-MB-231 and Hs578T cells was verified by Western blotting analysis (Fig. 4a). Intriguingly, both GPD1L and CYGB overexpression can significantly reduce the glycolytic metabolism of MDA-MB-231 and Hs578T cells, as revealed by reduced glucose uptake (Fig. 4b), lactate production (Fig. 4c), ECAR (Fig. 4d), and glycolytic capacity (Fig. 4e). These findings support a negative regulatory role of GPD1L and CYGB in the Warburg effect of TNBC cells.

### miR-210-3p confers growth-advantage and anti-apoptotic activity in TNBC by targeting GPD1L and CYGB

**Fig. 5:**
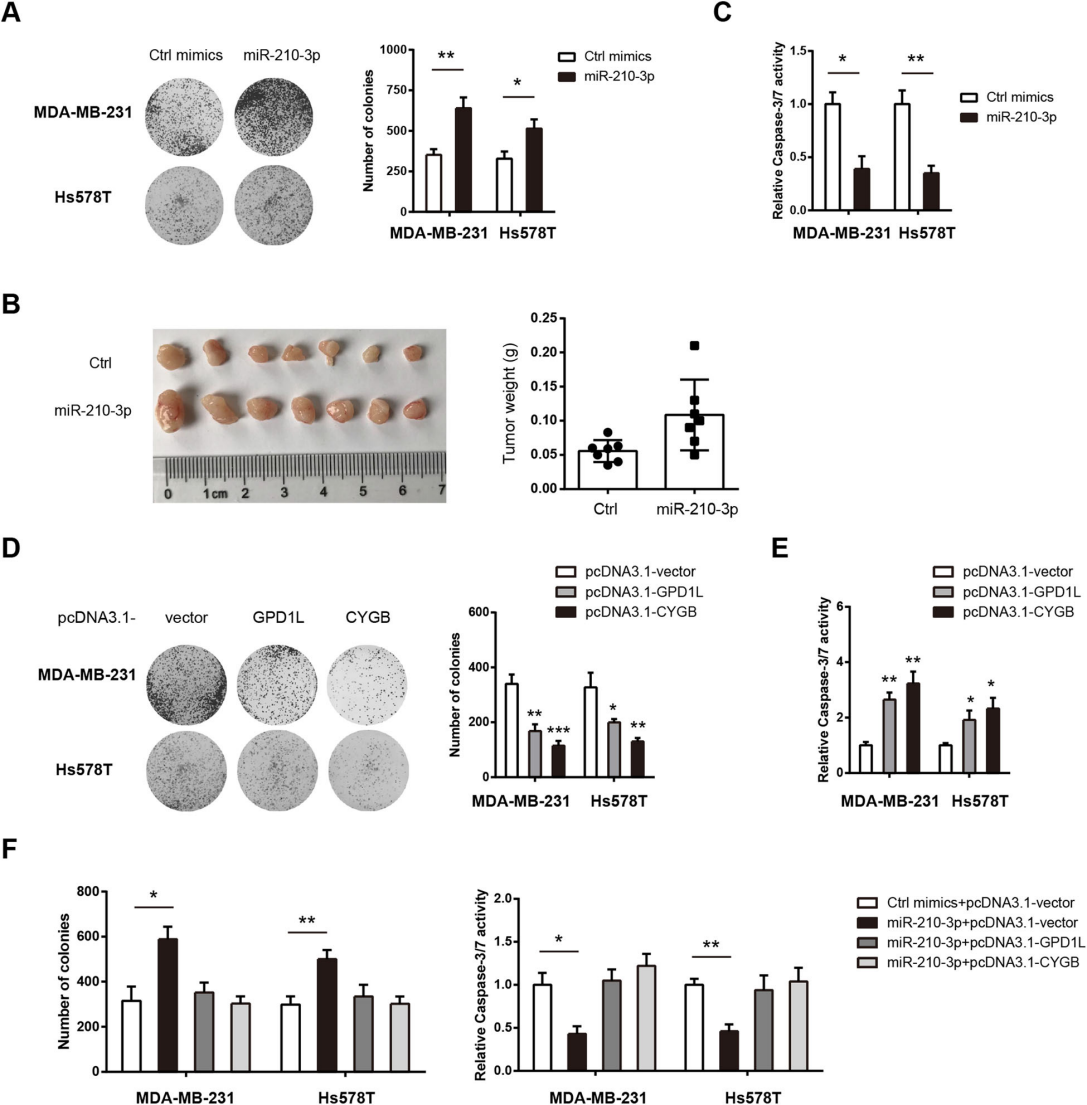
miR-210-3p confers growth-advantage and anti-apoptotic activity in TNBC by targeting GPD1L and CYGB. **a** The plate colony formation ability of MDA-MB-231 and Hs578T cells after transfection with control or miR-210-3p mimics. **b** The effect of miR-210-3p on in vivo tumor growth was studied by a subcutaneous xenograft model. **c** After serum starvation for 48 h, cell apoptosis in control or miR-210-3p mimic-transfected MDA-MB-231 and Hs578T cells was measured by Caspase-3/7 activity. **d** The plate colony formation ability of MDA-MB-231 and Hs578T cells after ectopic expression of GPD1L or CYGB. **e** After serum starvation for 24 h, cell apoptosis status in pcDNA3.1-vector, GPD1L-overexpressing or CYGB-overexpressing MDA-MB-231 and Hs578T cells was measured by Caspase-3/7 activity. **f** Measurement of the effects of miR-210-3p on cell proliferation and cell apoptosis upon overexpression of GPD1L or CYGB in MDA-MB-231 and Hs578T cells. *p < 0.05; **p < 0.01; ***p < 0.001.

To determine the potential oncogenic role of miR-210-3p in TNBC, we transfected MDA-MB-231 and Hs578T cells with miR-210-3p mimics or control mimics. CCK8 assay and plate colony formation assay showed that cell growth was promoted by miR-210-3p (Fig. 5a and Supplementary Fig. 1A). By using a subcutaneous xenograft model, we confirmed the growth-promoting effect of miR-210-3p in vivo (Fig. 5b). Apo-ONE Caspase-3/7 apoptosis assay revealed that miR-210-3p attenuated Caspase-3/7 activity in MDA-MB-231 and Hs578T cells (Fig. 5c). In contrast, loss-of-function studies showed that inhibition of miR-210-3p inhibited cell proliferation and promoted cell apoptosis (Supplementary Fig. 1B–D). Expectedly, overexpression of GPD1L or CYGB remarkably inhibited the cell viability and colony formation ability of MDA-MB-231 and Hs578T cells (Fig. 5c and Supplementary Fig. 1E). Expectedly, starvation-induced cell apoptosis was increased after ectopic expression of GPD1L or CYGB in MDA-MB-231 and Hs578T cells (Fig. 5d). Next, we examined whether miR-210-3p regulates TNBC cell proliferation and apoptosis through suppressing GPD1L or CYGB. Interestingly, ectopic expression of GPD1L or CYGB can largely compromise the growth-advantage induced by miR-210-3p; likewise, miR-210-3p-mediated anti-apoptotic activity was also blocked by GPD1L or CYGB overexpression (Fig. 5e). Taken together, GPD1L and CYGB are functional mediators of miR-210-3p in TNBC.

### miR-210-3p modulates HIF-1α and p53 activity via GPD1L and CYGB

**Fig. 6:**
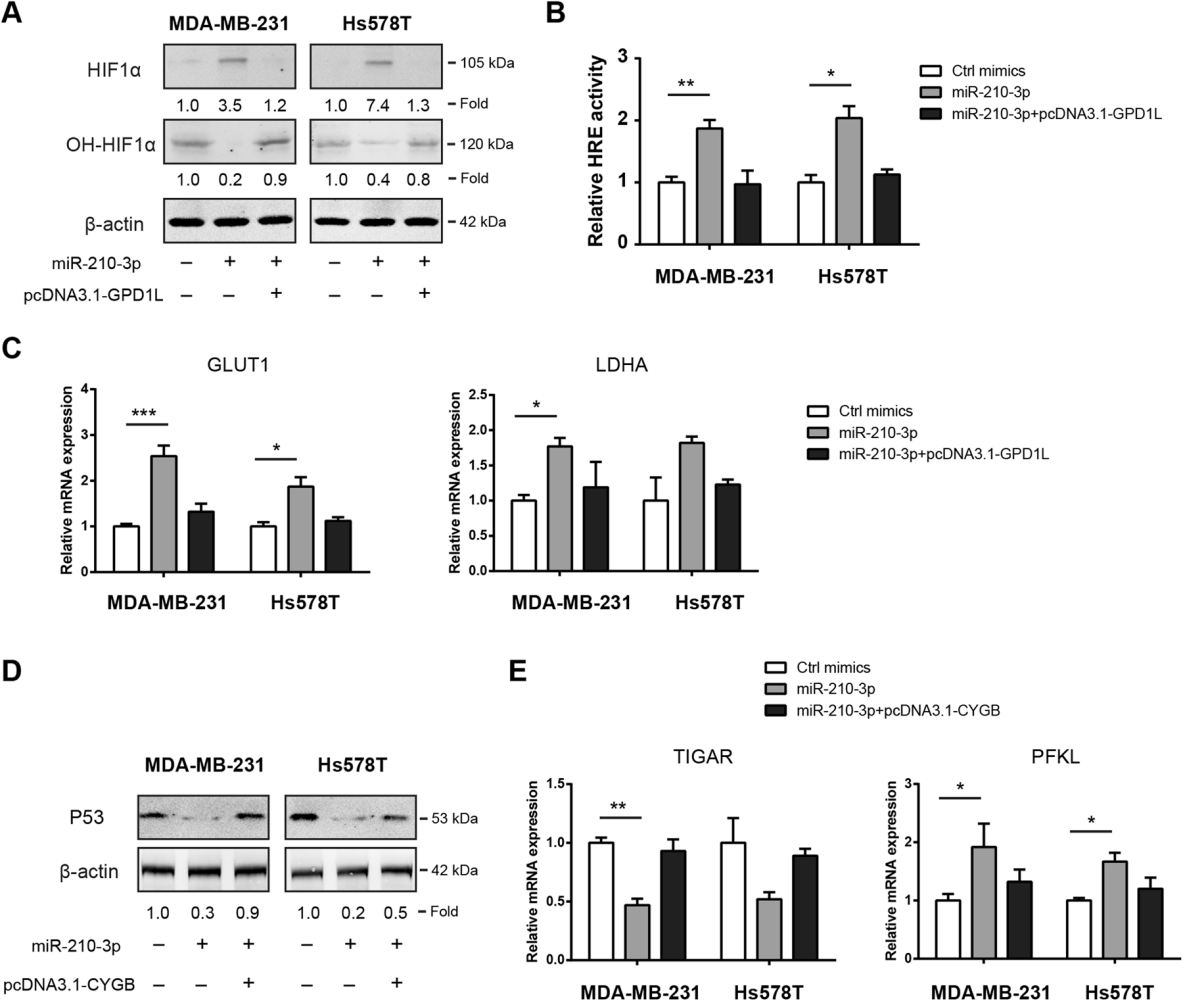
miR-210-3p modulates HIF1α and p53 activity via GPD1L and CYGB. **a** Western blotting analysis of the effect of miR-210-3p/GPD1L axis on HIF1α expression in MDA-MB-231 and Hs578T cells. **b** The effect of miR-210-3p/GPD1L axis on HIF1α transcriptional activity in MDA-MB-231 and Hs578T cells was detected by luciferase reporter experiment. **c** Real-time qPCR analysis of the effect of miR-210-3p/GPD1L axis on GLUT1 and LDHA mRNA expression in MDA-MB-231 and Hs578T cells. **d** Western blotting analysis of the effect of miR-210-3p/CYGB axis on p53 expression in MDA-MB-231 and Hs578T cells. **e** Real-time qPCR analysis of the effect of miR-210-3p/CYGB axis on TIGAR and PFKL mRNA expression in MDA-MB-231 and Hs578T cells. *p < 0.05; **p < 0.01; ***p < 0.001.

**Fig. 7:**
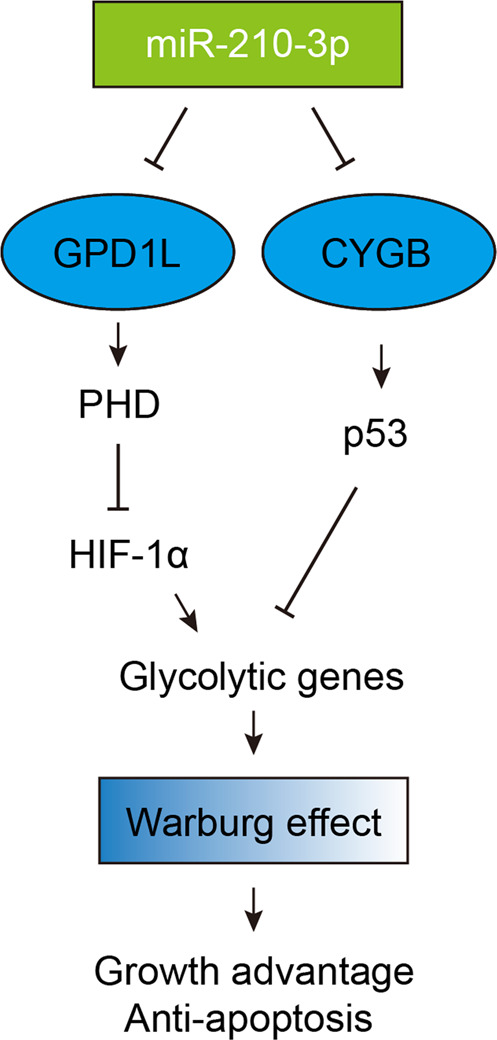
Proposed model illustrating that miR-210-3p promotes the Warburg effect to facilitate tumor growth and avoid cell apoptosis. On the one hand, miR-210-3p targets GPD1L, which is known to maintain prolyl hydroxylase (PHD) activity; PHD is essential to hydroxylate pralines HIF-1α and leads to its degradation by the proteasome. On the other hand, miR-210-3p targets CYGB, which stabilize p53 in TNBC. Ultimately, miR-210-3p contributes to increased HIF-1α activity and decreased p53 activity in TNBC.

Previously, the enzyme glycerol-3-phosphate dehydrogenase 1-like (GPD1L) has been demonstrated to increase prolyl hydroxylase (PHD) activity and decrease HIF-1α activity^21^. HIF-1α is a key transcriptional regulator of aerobic glycolysis by targeting several glucose transporter and glycolytic enzymes^22^. We found that miR-210-3p reduced hydroxy-HIF-1α level, suggesting the regulatory role of miR-210-3p on PHD activity. Moreover, miR-210-3p promoted HIF-1α protein accumulation and this phenomenon was disappeared after ectopic expression of GPD1L (Fig. 6a). Luciferase reporter assay showed that endogenous HIF-1α transcriptional activity was increased by miR-210-3p but restored to basal level after overexpression of GPD1L in MDA-MB-231 and Hs578T cells (Fig. 6b). In addition, the expression of two known HIF-1α targets, GLUT1 and LDHA, was tightly regulated by the miR-210-3p/GPD1L axis (Fig. 6c). Cytoglobin (CYGB) is downregulated in a number of malignancies and act as a tumor suppressor^23^. Notably, CYGB is able to stabilize p53 in osteosarcoma^24^. Given that p53 is known regulator of glucose metabolism, we examined if miR-210-3p/CYGB axis modulates the Warburg effect through p53. Indeed, p53 protein expression was decreased by treatment with miR-210-3p mimics and overexpression of CYGB can block the inhibitory effect of miR-210-3p on p53 activity (Fig. 6d). Meanwhile, the mRNA expression of TIGAR and PFKL, two glucose metabolism regulators downstream of p53, was also affected by the miR-210-3p/CYGB axis (Fig. 6e). Taken together, miR-210-3p can increase HIF-1α activity and suppress p53 activity via targeting GPD1L and CYGB, respectively. This leads to enhanced Warburg effect, which ultimately facilitate tumor growth and avoid cell apoptosis in TNBC (Fig. 7).

## Discussion

MicroRNAs are demonstrated to be critical modulators in the development and progression of cancers. In this study, we provide new evidence regarding the contribution of miRNAs in tumor glycolytic metabolism. Specifically, miR-210-3p was highly expressed in TNBC and was identified as a tumor promoter. Ectopic expression of miR-210-3p promoted the glycolytic phenotype and malignant properties of TNBC cells. Further mechanism study revealed that miR-210-3p modulates HIF-1α and p53 activity via directly targeting GPD1L and CYGB, resulting in enhanced aerobic glycolysis. Our results shed lights on the critical functions of miRNAs in TNBC glucose metabolism and suggest that miR-210-3p regulon might be a novel target for TNBC prevention and therapy.

Previously, several microRNAs have been demonstrated to play a role in the Warburg effect in breast cancer. For example, miR-155 promotes aerobic glycolysis via PIK3R1-FOXO3a-cMYC axis in breast cancer^18^; miR-342-3p inhibits oncogenic metabolic reprogramming via targeting monocarboxylate transporter 1 in TNBC^25^. Here, we further broadened this data through a large-scale screening study. Our results showed that miR-210-3p, miR-105-5p, and miR-767-5p are potential glycolytic regulators. The expression pattern and cellular roles of miR-210-3p has been reported in a several types of human cancers. Interestingly, both tumor-promoting and tumor-suppressive roles of miR-210-3p have been revealed. In prostate cancer, miR-210-3p maintains the activation of NF-κB signaling via targeting TNIP1 and SOCS1, resulting in EMT, invasion, migration, and bone metastasis of tumor cells^26^. Similarly, exosome-mediated transfer of miR-210-3p promotes lung cancer cell invasion by activating STAT3 signaling-induced EMT^27^. In colon cancer, miR-210-3p was reported to sustain DNA damage repair by metabolic adaptation^28^. Moreover, miR-210-3p is predictive of clear-cell renal cell carcinoma recurrence, pointing to potential utility as biomarkers^29^. In line with our findings, Li et al. showed that miR-210-3p expression level is significantly higher in breast cancer patients compared with normal controls^30^. Additionally, a close association between higher levels of miR-210-3p and risk of breast cancer progression (HR: 2.13, 95%CI: 1.33–3.39, *P* = 0.002) was found^31^. In the current study, we uncovered a novel function of miR-210-3p in regulating the glycolytic phenotypes of TNBC. Increased glycolysis provides sufficient cellular buildings for rapid cell proliferation^5^, and ultimately contributes to the oncogenic roles of miR-210-3p in TNBC.

To purse the molecular mechanism by which miR-210-3p promotes aerobic glycolysis in TNBC, we identified two functional mediators GPD1L and CYGB. GPD1L acts as tumor suppressor in cancers^32,33^; one mechanism is to inhibit the activity of prolyl hydroxylase (PHD), which hydroxylates prolines in HIF-1α and promotes its proteasome degradation^21,33^. Consistently, we revealed that HIF-1α protein expression and HRE activity is significantly increased by miR-210-3p, and suppressed after ectopic expression of GPD1L. Interestingly, miR-210 is a direct transcriptional target of HIF-1α, indicating a positive feedback loop between miR-210 and HIF-1α^21^. Previously, several studies have demonstrated a tumor-suppressive role of CYGB in cancers, especially in breast cancer. CYGB is downregulated in many human cancers due to promoter hypermethylation. Mechanistically, CYGB might exhibit anti-tumor effect by ROS scavenging^23^. Despite Hs578T and MDA-MB-231 cell lines express V157F and R280K p53 mutants^34^, consistent with our findings, CYGB inhibits metabolic reprogramming in a p53-dependent manner in breast cancer^35^. Apart from GPD1L and CYGB, miR-210-3p can target several other genes in cancers, such as FGFRL1^36^, SIN3A^37^, and EphrinA3^38^. This may explain why miR-210-3p exhibit both tumor suppressor gene and oncogene features in different cancers. In addition, we cannot fully rule out the contribution of other targets of miR-210-3p in regulating the glycolytic phenotype of TNBC.

In conclusion, miR-210-3p acts as a key regulon in metabolic glucose reprogramming of TNBC. Molecular mechanism revealed that GPD1L and CYGB are direct downstream targets of miR-210-3p-mediated aerobic glycolysis and oncogenic activities. Altogether, our data provides essential insights into miRNA regulation of TNBC glycolysis and opens potential avenues for targeting miR-210-3p in clinical treatment of TNBC.

## Supplementary information

Supplemenary Table 1

Supplementary Figure 1

Supplementary Figure Legend
