# Supplementary material for: A miR-210-3p regulon that controls the Warburg effect by modulating HIF-1α and p53 activity in triple-negative breast cancer
Source: Cell Death Dis. 2020 Sep 9;11(9):731. doi: 10.1038/s41419-020-02952-6 (PMC7481213; doi:10.1038/s41419-020-02952-6)
Supplement: Supplementary file 1 — Supplemenary Table 1 [file 41419_2020_2952_MOESM1_ESM.docx]

**Supplementary Table 1. Group information**

| Sample ID | group |
| --- | --- |
| TCGA-A1-A0SP-01 | Gly-high |
| TCGA-A2-A0CM-01 | Gly-high |
| TCGA-A2-A0D0-01 | Gly-high |
| TCGA-A2-A0D2-01 | Gly-high |
| TCGA-A2-A0T0-01 | Gly-high |
| TCGA-A2-A0T2-01 | Gly-high |
| TCGA-A2-A3XT-01 | Gly-high |
| TCGA-A2-A3XX-01 | Gly-high |
| TCGA-A7-A0DA-01 | Gly-high |
| TCGA-A7-A4SE-01 | Gly-high |
| TCGA-A7-A6VV-01 | Gly-high |
| TCGA-A7-A6VW-01 | Gly-high |
| TCGA-A7-A6VY-01 | Gly-high |
| TCGA-A8-A07O-01 | Gly-high |
| TCGA-A8-A09X-01 | Gly-high |
| TCGA-AC-A2QH-01 | Gly-high |
| TCGA-AC-A2QJ-01 | Gly-high |
| TCGA-AC-A7VC-01 | Gly-high |
| TCGA-AN-A0AL-01 | Gly-high |
| TCGA-AN-A0AT-01 | Gly-high |
| TCGA-AN-A0G0-01 | Gly-high |
| TCGA-AN-A0XU-01 | Gly-high |
| TCGA-AO-A0J4-01 | Gly-high |
| TCGA-AO-A0J6-01 | Gly-high |
| TCGA-AO-A124-01 | Gly-high |
| TCGA-AO-A129-01 | Gly-high |
| TCGA-AR-A0U4-01 | Gly-high |
| TCGA-AR-A1AY-01 | Gly-high |
| TCGA-AR-A5QQ-01 | Gly-high |
| TCGA-B6-A400-01 | Gly-high |
| TCGA-B6-A402-01 | Gly-high |
| TCGA-BH-A0B3-01 | Gly-high |
| TCGA-BH-A0B9-01 | Gly-high |
| TCGA-BH-A0E0-01 | Gly-high |
| TCGA-BH-A0WA-01 | Gly-high |
| TCGA-BH-A18G-01 | Gly-high |
| TCGA-BH-A1F6-01 | Gly-high |
| TCGA-C8-A131-01 | Gly-high |
| TCGA-C8-A26X-01 | Gly-high |
| TCGA-C8-A26Y-01 | Gly-high |
| TCGA-C8-A27B-01 | Gly-high |
| TCGA-D8-A13Z-01 | Gly-high |
| TCGA-D8-A143-01 | Gly-high |
| TCGA-D8-A1XK-01 | Gly-high |
| TCGA-D8-A1XQ-01 | Gly-high |
| TCGA-D8-A27F-01 | Gly-high |
| TCGA-D8-A27H-01 | Gly-high |
| TCGA-E2-A14N-01 | Gly-high |
| TCGA-E2-A150-01 | Gly-high |
| TCGA-E2-A1LL-01 | Gly-high |
| TCGA-E9-A5FL-01 | Gly-high |
| TCGA-EW-A1P4-01 | Gly-high |
| TCGA-EW-A1P8-01 | Gly-high |
| TCGA-EW-A1PB-01 | Gly-high |
| TCGA-GM-A2DB-01 | Gly-high |
| TCGA-OL-A6VO-01 | Gly-high |
| TCGA-S3-AA10-01 | Gly-high |
| TCGA-A1-A0SK-01 | Gly-low |
| TCGA-A2-A04U-01 | Gly-low |
| TCGA-A2-A0SX-01 | Gly-low |
| TCGA-A2-A1G6-01 | Gly-low |
| TCGA-A2-A3XY-01 | Gly-low |
| TCGA-A7-A26G-01 | Gly-low |
| TCGA-A8-A07C-01 | Gly-low |
| TCGA-A8-A08R-01 | Gly-low |
| TCGA-AC-A2BK-01 | Gly-low |
| TCGA-AC-A6IW-01 | Gly-low |
| TCGA-AN-A04D-01 | Gly-low |
| TCGA-AN-A0AR-01 | Gly-low |
| TCGA-AO-A03U-01 | Gly-low |
| TCGA-AO-A0JL-01 | Gly-low |
| TCGA-AO-A128-01 | Gly-low |
| TCGA-AO-A12F-01 | Gly-low |
| TCGA-AO-A1KR-01 | Gly-low |
| TCGA-AQ-A04J-01 | Gly-low |
| TCGA-AR-A0TS-01 | Gly-low |
| TCGA-AR-A1AR-01 | Gly-low |
| TCGA-AR-A256-01 | Gly-low |
| TCGA-AR-A2LR-01 | Gly-low |
| TCGA-B6-A3ZX-01 | Gly-low |
| TCGA-BH-A0BG-01 | Gly-low |
| TCGA-BH-A0BL-01 | Gly-low |
| TCGA-BH-A0RX-01 | Gly-low |
| TCGA-BH-A18V-01 | Gly-low |
| TCGA-BH-A1EW-01 | Gly-low |
| TCGA-BH-A1FC-01 | Gly-low |
| TCGA-BH-A42U-01 | Gly-low |
| TCGA-C8-A12V-01 | Gly-low |
| TCGA-C8-A1HJ-01 | Gly-low |
| TCGA-C8-A3M7-01 | Gly-low |
| TCGA-D8-A147-01 | Gly-low |
| TCGA-D8-A1JF-01 | Gly-low |
| TCGA-D8-A1JL-01 | Gly-low |
| TCGA-D8-A27M-01 | Gly-low |
| TCGA-E2-A14R-01 | Gly-low |
| TCGA-E2-A14X-01 | Gly-low |
| TCGA-E2-A158-01 | Gly-low |
| TCGA-E2-A1L7-01 | Gly-low |
| TCGA-E2-A1LH-01 | Gly-low |
| TCGA-E2-A1LS-01 | Gly-low |
| TCGA-EW-A1OV-01 | Gly-low |
| TCGA-EW-A1OW-01 | Gly-low |
| TCGA-EW-A1PH-01 | Gly-low |
| TCGA-EW-A3U0-01 | Gly-low |
| TCGA-EW-A6SB-01 | Gly-low |
| TCGA-GI-A2C9-01 | Gly-low |
| TCGA-GM-A2DF-01 | Gly-low |
| TCGA-GM-A2DH-01 | Gly-low |
| TCGA-HN-A2NL-01 | Gly-low |
| TCGA-LL-A441-01 | Gly-low |
| TCGA-LL-A5YO-01 | Gly-low |
| TCGA-LL-A73Y-01 | Gly-low |
| TCGA-S3-AA15-01 | Gly-low |
