# Supplementary figures and images for: A miR-210-3p regulon that controls the Warburg effect by modulating HIF-1α and p53 activity in triple-negative breast cancer
Source: Cell Death Dis. 2020 Sep 9;11(9):731. doi: 10.1038/s41419-020-02952-6 (PMC7481213; doi:10.1038/s41419-020-02952-6)

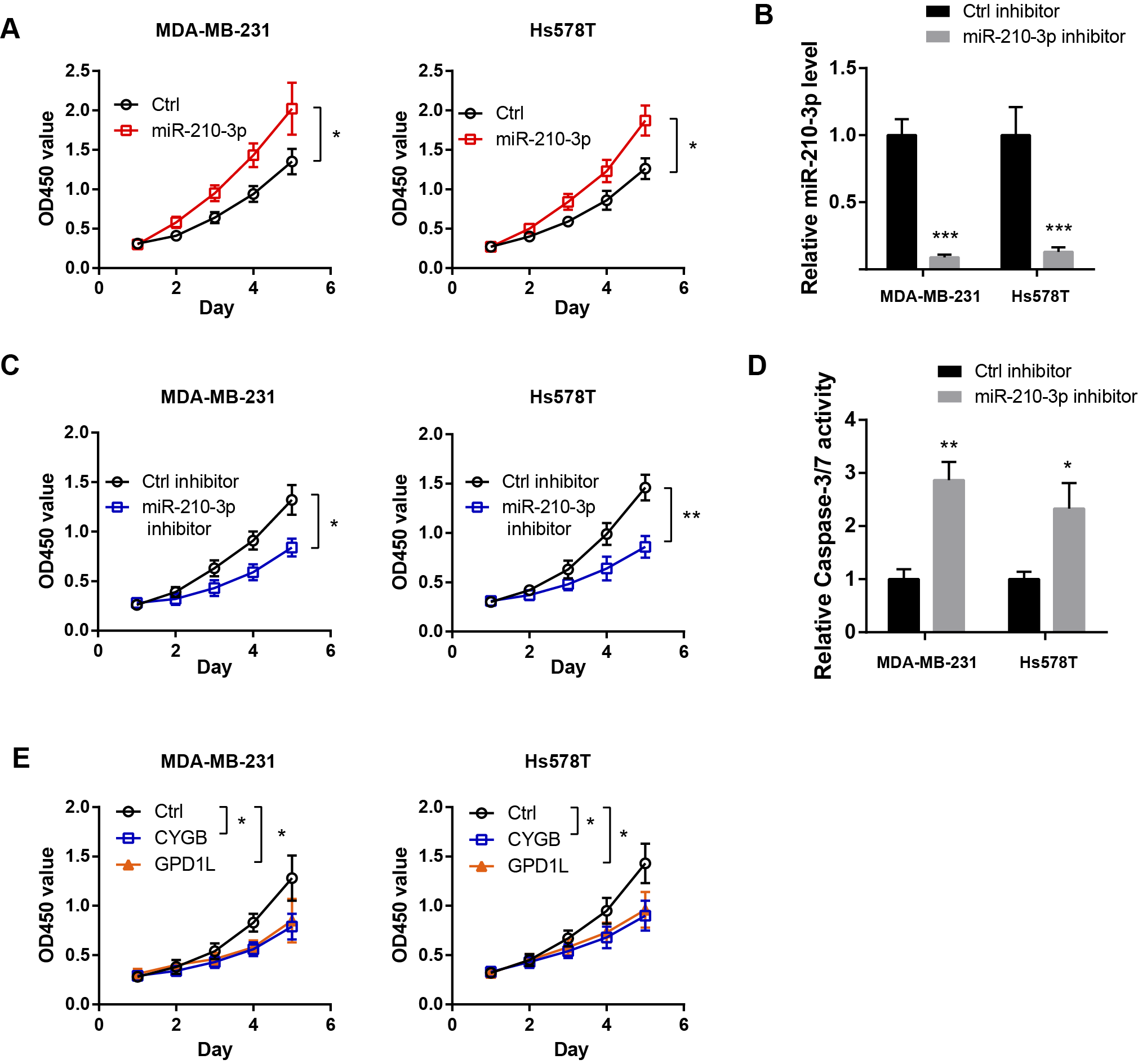

Supplement: Supplementary file 2 — Supplementary Figure 1 [file 41419_2020_2952_MOESM2_ESM.tif]
