# Supplementary material for: A miR-210-3p regulon that controls the Warburg effect by modulating HIF-1α and p53 activity in triple-negative breast cancer
Source: Cell Death Dis. 2020 Sep 9;11(9):731. doi: 10.1038/s41419-020-02952-6 (PMC7481213; doi:10.1038/s41419-020-02952-6)
Supplement: Supplementary file 3 — Supplementary Figure Legend [file 41419_2020_2952_MOESM3_ESM.docx]

**Supplementary Figure 1** miR-210-3p confers growth-advantage and anti-apoptotic activity in TNBC cells. (A) The cell viability of MDA-MB-231 and Hs578T cells after transfection with control or miR-210-3p mimics. (B) The inhibition efficiency of miR-210-3p in MDA-MB-231 and Hs578T cells. (C) The cell viability of MDA-MB-231 and Hs578T cells after transfection with control or miR-210-3p inhibitors. (D) After serum starvation for 48 h, cell apoptosis in control or miR-210-3p inhibitor-transfected MDA-MB-231 and Hs578T cells was measured by Caspase-3/7 activity. (E) Cell proliferation in pcDNA3.1-vector, GPD1L-overexpressing or CYGB-overexpressing MDA-MB-231 and Hs578T cells was measured by CCK-8 assay. *: p < 0.05; **: p < 0.01; ***: p < 0.001.
